# Glucocorticoid-Sparing Effect of Biologic Therapy in Rheumatoid Arthritis: A Single-Center Longitudinal Observational Study Comparing Rituximab With Tumor Necrosis Factor Inhibitors

**DOI:** 10.7759/cureus.113210

**Published:** 2026-07-23

**Authors:** Ilham Ben Marzouk, Imane El Binoune, Samira Rostom, Ikram EL Moubarik, Salma Zemrani, Bouchra AMINE, Kaoutar Dib, Rachid Bahiri

**Affiliations:** 1 Rheumatology A Department, El Ayachi Hospital, Ibn Sina University Hospital, Rabat-Sale, MAR; 2 Laboratory of Biostatistics, Clinical Research and Epidemiology (LBRCE), Mohammed V University in Rabat, Rabat, MAR

**Keywords:** anti-tnf, biologic therapy, glucocorticoids, glucocorticoid-sparing, rheumatoid arthritis, rituximab

## Abstract

Introduction: Glucocorticoids (GCs) remain an important component of rheumatoid arthritis (RA) management because of their rapid anti-inflammatory effects. However, prolonged exposure to GCs is associated with significant adverse events. Current recommendations advocate minimizing GC use through rapid tapering and discontinuation whenever clinically feasible. This study aimed to evaluate the GC-sparing effect of biologic therapy in patients with RA and to compare outcomes between rituximab and anti-TNF agents. Materials and methods: This longitudinal, observational, single-center study included adult patients who fulfilled the 2010 American College of Rheumatology/European League Against Rheumatism (ACR/EULAR) classification criteria for RA and started rituximab or anti-TNF therapy while receiving long-term GC treatment for more than three months at a prednisone-equivalent dose greater than 5 mg/day. Patients were assessed at baseline and after four and six months of treatment for GC dose, disease activity (Disease Activity Score in 28 joints based on CRP (DAS28-CRP)), inflammatory markers (CRP and ESR), and functional disability (Health Assessment Questionnaire (HAQ)). The primary outcome was the proportion of patients achieving a GC dose ≤5 mg/day at six months. Secondary outcomes included mean GC doses at four and six months, complete GC withdrawal, and comparison of GC dose reduction between treatment groups. Results: Sixty patients were included, with a mean age of 52.6 ± 11.6 years, and 58 (96.7%) were female. Twenty-nine patients received rituximab, and 31 received anti-TNF therapy. The mean GC dose decreased significantly from 8.74 ± 1.93 mg/day at baseline to 7.11 ± 2.28 mg/day at four months and 6.07 ± 2.07 mg/day at six months (F = 53.4, p < 0.001). At six months, 31 patients (51.7%) achieved a GC dose ≤5 mg/day, whereas complete GC withdrawal was achieved in only one patient (1.7%). Baseline DAS28-CRP was significantly lower in patients who achieved a GC dose ≤5 mg/day at six months than in those who remained on >5 mg/day (4.82 ± 0.77 vs. 5.36 ± 0.96, t = 2.44, p = 0.02). GC doses decreased significantly over time, with significant differences across all study visits (all post hoc comparisons, p < 0.01). No significant differences were observed between the rituximab and anti-TNF groups in GC doses at baseline, four months, or six months, or in the mean GC dose reduction from baseline to six months (−2.55 ± 1.70 vs. −2.77 ± 2.40 mg/day, t = 0.41, p = 0.68). Conclusions: Biologic therapy was associated with a significant GC-sparing effect in patients with RA, with a progressive reduction in GC exposure during the first six months of treatment. More than half of the patients achieved a prednisone-equivalent dose ≤5 mg/day, although complete GC withdrawal remained uncommon. No superiority of rituximab over anti-TNF agents was observed regarding the GC-sparing effect. Given the relatively small sample size (n = 60), larger prospective multicenter studies are warranted to confirm these findings.

## Introduction

Rheumatoid arthritis (RA) is a chronic autoimmune disease characterized by recurrent episodes of joint inflammation and systemic manifestations. Despite major advances in biologic therapies, glucocorticoids (GCs) remain widely used because of their rapid anti-inflammatory effects and their role as bridging therapy during the initiation or adjustment of disease-modifying antirheumatic drugs (DMARDs). However, prolonged exposure to GCs is associated with substantial morbidity, including osteoporosis, cardiovascular disease, diabetes mellitus, and an increased risk of infections [1,2].

The 2022 update of the European League Against Rheumatism (EULAR) recommendations strongly emphasizes that GCs should be prescribed for the shortest duration possible, particularly during the early phase of conventional synthetic DMARD (csDMARD) initiation, and tapered as rapidly as clinically feasible [3]. In patients achieving sustained remission, as assessed by validated composite measures of disease activity (Disease Activity Score in 28 joints (DAS28)), GCs should be discontinued before considering tapering of biologic DMARDs (bDMARDs) or csDMARDs [3].

Despite these recommendations, discontinuation of GCs remains challenging in routine clinical practice. Real-world studies have shown that nearly half of patients treated with bDMARDs, including tumor necrosis factor inhibitors (anti-TNF) and rituximab, continue low-dose prednisone for more than six months after biologic initiation [4-6]. Moreover, the commonly accepted threshold defining “low-dose” GC therapy (≤7.5 mg/day) remains largely arbitrary and does not correspond to a clearly established threshold of efficacy or safety [7].

GC tapering is further complicated by the unpredictable risk of disease flare, which may occur even in patients with sustained disease control [8,9]. Although approximately 50% of patients with early RA can discontinue GCs within three years, up to 30% experience disease flare during the first six months following withdrawal [10]. These findings emphasize the need to better define GC tapering strategies and to identify predictors of successful GC withdrawal, particularly in patients receiving biologic therapies such as rituximab or anti-TNF agents.

A daily prednisone dose of 5 mg represents a clinically meaningful threshold in RA and is frequently used as a maintenance dose in patients with controlled disease. The SEMIRA trial [11] showed that maintaining prednisone at 5 mg/day provided better disease control than complete GC withdrawal. In contrast, the GLORIA trial [12] demonstrated that even this low dose remained associated with treatment-related adverse events. Therefore, achieving a prednisone dose of ≤5 mg/day may be considered a pragmatic GC-sparing target while recognizing that complete withdrawal remains the ultimate goal. TNF inhibitors and rituximab are among the most widely used biologic therapies for RA. However, few studies have directly compared their GC-sparing effects.

The present study aimed to evaluate GC tapering in patients with RA initiating biologic therapy and to assess the effectiveness of biologic agents in achieving GC-sparing outcomes in routine clinical practice.

## Materials and methods

Study design

This longitudinal, observational, single-center study was conducted at a tertiary rheumatology department in Morocco. Medical records of patients with RA who initiated biologic therapy between June 2025 and December 2025 were reviewed. Eligible patients had persistent active RA despite treatment with csDMARDs and initiated biologic therapy according to EULAR recommendations and the treating rheumatologist's clinical judgment, regardless of the treatment line. Patients were followed for six months after biologic initiation.

Inclusion and exclusion criteria

Eligible patients were adults aged ≥18 years fulfilling the 2010 ACR/EULAR classification criteria for RA and initiating treatment with a bDMARD, namely either a tumor necrosis factor inhibitor (anti-TNF) or rituximab. Only patients receiving long-term GC therapy for more than three months, with a daily prednisone-equivalent dose >5 mg at the time of biologic initiation, were included.

Patients were excluded in cases of refusal to participate, irregular follow-up during the six-month study period, cognitive impairment, age <18 years, or absence of GC therapy at baseline.

Ethical approval

The study was conducted in accordance with the principles of the Declaration of Helsinki and was approved by the Ethics Committee for Biomedical Research of Mohammed V University (approval number: 66-26).

Data collection

The following data were collected for all participants: socio-demographic characteristics, including age and sex; medical history, including hypertension, diabetes mellitus, dyslipidemia, cardiovascular disease, osteoporosis, fibromyalgia, and other comorbidities; and disease-related characteristics, including diagnostic delay, disease duration, rheumatoid factor (RF) and anti-cyclic citrullinated peptide (anti-CCP) antibody status, erosive disease, extra-articular manifestations, and disease activity, which was assessed using the DAS28 based on CRP (DAS28-CRP) and erythrocyte sedimentation rate (DAS28-ESR).

Therapeutic characteristics: previous or concomitant use of csDMARDs, analgesics, prior exposure to biologic therapies, type of biologic therapy initiated, baseline GC dose, and GC exposure, assessed by recording the daily prednisone-equivalent dose (mg/day) at each assessment time point. Disease activity and GC exposure were assessed at current biologic initiation (M0) and at four months (M4) and six months (M6) after treatment initiation.

Outcomes

The primary outcome was the proportion of patients achieving a daily GC dose ≤5 mg at six months after biologic therapy initiation. Secondary outcomes included the mean daily GC dose at M4 and M6, the proportion of patients who completely discontinued GC therapy during the six-month follow-up, and the comparison of GC-sparing effects between patients treated with rituximab and those treated with anti-TNF agents.

Statistical analysis

Statistical analyses included descriptive and comparative analyses and were performed using Jamovi software version 2.4.14 (The Jamovi Project Pty Ltd., Australia). Quantitative variables were expressed as mean ± standard deviation or median (interquartiles), according to data distribution assessed using the Shapiro-Wilk test. Categorical variables were expressed as frequencies and percentages.

Comparisons were performed using Student's t-test or the Mann-Whitney U test for continuous variables, and the chi-square test or Fisher's exact test for categorical variables, as appropriate. Changes in GC doses over time (M0, M4, and M6) were analyzed using repeated-measures analysis of variance (ANOVA). Test statistics (t, U, χ², or F, as applicable) were reported alongside p-values. A two-sided p-value < 0.05 was considered statistically significant.

## Results

A total of 60 patients with RA were included in the study. The baseline demographic, clinical, and therapeutic characteristics of the study population are summarized in Table 1.

**Table 1:** Baseline characteristics of the study population ¹ mean ± standard deviation, ² median (interquartile range), ³ number (percentage) Anti-CCP: anti-cyclic citrullinated peptide antibodies, CRP: C-reactive protein, csDMARDs: conventional synthetic disease-modifying antirheumatic drugs, DAS28-CRP: Disease Activity Score in 28 joints using C-reactive protein, DAS28-ESR: Disease Activity Score in 28 joints using erythrocyte sedimentation rate, HAQ: Health Assessment Questionnaire, SDAI: Simplified Disease Activity Index, VAS: visual analog scale

Variables	N = 60
Age (years)^1^	52.6 ± 11.6
Female^3^	58 (96.7)
Disease duration (years)^2^	15.5 (11.0-24.0)
Anti-CCP positivity ^3^	53 (88.3)
Erosive disease^3^	52 (86.7)
Hypertension^3^	10 (16.7)
Diabetes mellitus^3^	22 (36.7)
Fibromyalgia^3^	21 (35.0)
Osteoporosis^3^	41 (68.3)
HAQ^2^	1 (0.8-1.5)
Previous biologic therapy^3^	23 (38.3)
Concomitant csDMARDs	
None^3^	25 (41.7)
Methotrexate^3^	18 (30.0)
Leflunomide^3^	7 (11.7)
Sulfasalazine^3^	10 (16.7)
Baseline disease activity	
DAS28-ESR^1^	5.45 ± 0.84
DAS28-CRP^1^	5.08 ± 0.90
SDAI^1^	30.53 ± 12.16
ESR (mm/h)^2^	36.0 (21.5-64.3)
CRP (mg/L)^2^	20.9 (11.7-33.9)
Pain VAS^1^	6.22 ± 1.15
Current biologic therapy	
Rituximab^3^	29 (48.3)
Anti-TNF^3^	31 (51.7)
Type of anti-TNF agent (n = 31)	
Etanercept^3^	12 (38.7)
Infliximab^3^	6 (19.4)
Certolizumab pegol^3^	7 (22.6)
Golimumab^3^	6 (19.4)

The mean age was 52.6 ± 11.6 years, and the cohort was predominantly female (58 patients, 96.7%). Patients had long-standing disease, with a median disease duration of 15.5 years (11.0-24.0). Anti-CCP antibodies were positive in 53 patients (88.3%), and erosive disease was present in 52 patients (86.7%).

The most common comorbidities were osteoporosis (41 patients, 68.3%), diabetes mellitus (22 patients, 36.7%), and fibromyalgia (21 patients, 35.0%), whereas hypertension was reported in 10 patients (16.7%). The median Health Assessment Questionnaire (HAQ) score was 1.0 (0.8-1.5).

Regarding concomitant treatment, 35 patients (58.3%) received a csDMARD, most commonly methotrexate (18 patients, 30.0%), whereas 25 patients (41.7%) received biologic therapy (rituximab or a TNF inhibitor) without concomitant csDMARDs.

At M0, disease activity was high, with a mean DAS28-ESR of 5.45 ± 0.84, a mean DAS28-CRP of 5.08 ± 0.90, and a mean SDAI of 30.53 ± 12.16. The median ESR and CRP values were 36.0 mm/h (21.5-64.3) and 20.9 mg/L (11.7-33.9), respectively. The mean pain visual analog scale (VAS) score was 6.22 ± 1.15.

Previous exposure to biologic therapy was documented in 23 patients (38.3%). At biologic therapy initiation, 29 patients (48.3%) received rituximab, whereas 31 patients (51.7%) initiated anti-TNF therapy. Among patients receiving anti-TNF therapy, etanercept was the most frequently prescribed agent (12 patients, 38.7%), followed by certolizumab pegol (7 patients, 22.6%), infliximab (six patients, 19.4%), and golimumab (six patients, 19.4%).

Evolution of GC doses over time

The evolution of GC doses during follow-up is presented in Table 2. A progressive reduction in the mean daily GC dose was observed throughout the six-month follow-up period. The mean dose decreased from 8.74 ± 1.93 mg/day at M0 to 7.11 ± 2.28 mg/day at M4, and further to 6.07 ± 2.07 mg/day at M6. Repeated-measures ANOVA demonstrated a statistically significant reduction in mean GC doses over time (F = 53.4, p < 0.001). Post hoc pairwise comparisons using Tukey's post hoc test confirmed significant reductions between M0 and M4 (p < 0.01), between M0 and M6 (p < 0.001), and between M4 and M6 (p < 0.01). These findings indicate a gradual and sustained GC-sparing effect during the first six months following biologic therapy initiation (Table 2).

**Table 2:** Changes in GC dose over time (M0, M4, and M6) ¹ Mean ± standard deviation. Changes over time were analyzed using repeated-measures ANOVA (F = 53.4, p < 0.001). * Post hoc pairwise comparisons using Tukey's test showed statistically significant differences between all time points. M0: baseline, M4: month 4, M6: month 6, ANOVA: analysis of variance, GC: glucocorticoid

Variable	M0	M4	M6	Test used	Test statistic	p-value
GC dose (mg/day)^ 1^	8.74 ± 1.93*	7.11 ± 2.28*	6.07 ± 2.07*	Repeated-measures ANOVA	53.4	<0.001

**Figure 1:**
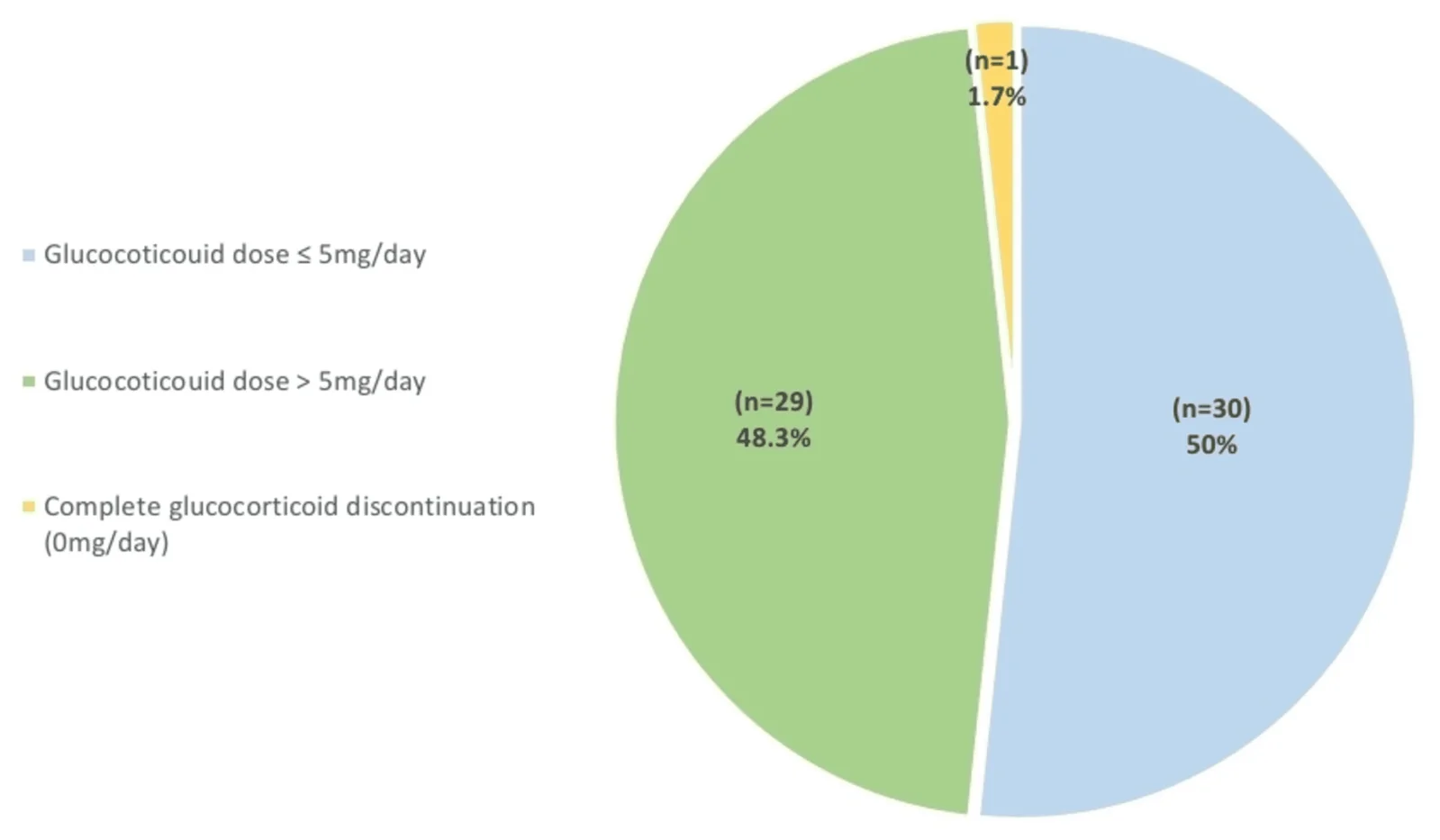
Distribution of GC doses at M6 following biologic therapy initiation GC: glucocorticoid, M6: month 6

At M4, 24 patients (40.0%) achieved a GC dose ≤5 mg/day. At M6, 31 patients (51.7%) achieved a GC dose ≤5 mg/day, including one patient (1.7%) who achieved complete GC withdrawal (0 mg/day), whereas 29 patients (48.3%) remained on a GC dose >5 mg/day (Figure 1).

For the comparative analysis presented in Table 3, baseline characteristics were compared between patients who achieved a GC dose ≤5 mg/day at M6 (n = 31) and those who remained on a dose >5 mg/day (n = 29).

**Table 3:** Comparison of baseline characteristics between patients achieving a GC dose of ≤5 mg/day and those Remaining on >5 mg/day at M6 Continuous variables (¹) are expressed as mean ± standard deviation and compared using Student's t-test. Categorical variables (2) are presented as n (%) and compared using the chi-square test with or without continuity correction, as appropriate. A p-value <0.05 was considered statistically significant. For this comparative analysis, the patient who completely discontinued GCs at M6 (0 mg/day) was included in the ≤5 mg/day group. Anti-CCP: anti-cyclic citrullinated peptide antibodies, DAS28-CRP: Disease Activity Score in 28 joints using C-reactive protein, DAS28-ESR: Disease Activity Score in 28 joints using erythrocyte sedimentation rate, HAQ: Health Assessment Questionnaire

Variable	≤5 mg/day (n = 31)	>5 mg/day (n = 29)	Test used	Test statistic	p-value
Age, years^1^	52.48 ± 10.11	52.83 ± 13.19	Student's t-test	0.11	0.91
Disease duration, years^1^	17.98 ± 8.12	16.90 ± 10.45	Student's t-test	0.45	0.66
Hypertension^2^	5 (16.1)	5 (17.2)	Chi² continuity correction	0.00	1.00
Osteoporosis^2^	23 (74.2)	18 (62.1)	Chi²	1.02	0.31
Previous biologic therapy^2^	11 (35.5)	12 (41.4)	Chi²	0.22	0.64
Fibromyalgia^2^	10 (32.3)	11 (37.9)	Chi²	0.21	0.64
Anti-CCP positivity^2^	27 (87.1)	26 (89.7)	Chi² continuity correction	0.00	1.00
Erosive disease^2^	26 (83.9)	26 (89.7)	Chi² continuity correction	0.08	0.78
DAS28-CRP^1^	4.82 ± 0.77	5.36 ± 0.96	Student's t-test	2.44	0.02
DAS28-ESR^1^	5.26 ± 0.93	5.65 ± 0.69	Student's t-test	1.84	0.07
HAQ^1^	1.06 ± 0.66	1.22 ± 0.56	Student's t-test	1.02	0.31

The two groups were comparable in terms of age and disease duration. Mean disease duration was 17.98 ± 8.12 years in the ≤5 mg/day group and 16.90 ± 10.45 years in the >5 mg/day group (Student's t-test, t = 0.45, p = 0.66). Fibromyalgia was present in 10 (32.3%) patients in the anti-TNF group and 11 (37.9%) patients in the rituximab group, with no significant difference between the two treatment groups (χ² = 0.21, p = 0.64). Baseline functional disability, assessed using the HAQ, was also comparable between the two groups (1.06 ± 0.66 vs. 1.22 ± 0.56; Student's t-test, t = 1.02, p = 0.31). However, baseline DAS28-CRP was significantly lower in patients who achieved a GC dose ≤5 mg/day than in those who remained on a dose >5 mg/day (4.82 ± 0.77 vs. 5.36 ± 0.96; Student's t-test, t = 2.44, p = 0.02) (Table 3).

Comparison of GC doses between treatment groups

GC doses were compared between patients treated with rituximab and those receiving anti-TNF therapy at each time point using Student's t-test. At M0, the mean daily GC dose was 8.36 ± 1.21 mg/day in the rituximab group and 9.10 ± 2.39 mg/day in the anti-TNF group, with no statistically significant difference between groups (t = −1.52, p = 0.13).

At M4, the mean daily GC dose decreased to 6.81 ± 1.88 mg/day in the rituximab group and 7.39 ± 2.61 mg/day in the anti-TNF group, with no statistically significant difference between groups (t = −0.97, p = 0.33). At M6, the mean daily GC dose further decreased to 5.81 ± 1.72 mg/day in the rituximab group and 6.33 ± 2.38 mg/day in the anti-TNF group, again with no statistically significant difference between groups (t = −0.94, p = 0.34). The mean change in GC dose from M0 to M6 (ΔGC dose, M6 − M0) was −2.55 ± 1.70 mg/day in the rituximab group and −2.77 ± 2.40 mg/day in the anti-TNF group, with no statistically significant difference between groups (t = 0.41, p = 0.68).

Overall, both treatment groups showed a reduction in GC exposure over time, with no significant difference in the GC-sparing effect between rituximab and anti-TNF therapy during the six-month follow-up period (Table 4).

**Table 4:** Comparison of GC doses between the rituximab and anti-TNF groups Continuous variables (¹) are presented as mean ± standard deviation and compared using Student's t-test. Δ represents the change in GC dose between M0 and M6. A p-value <0.05 was considered statistically significant. anti-TNF: anti-tumor necrosis factor, GC: glucocorticoid, M0: baseline, M6: month 6

Variable	Rituximab group	Anti-TNF group	Test used	Test statistic	p-value
GC dose (mg/day)^1 ^M0	8.36 ± 1.21	9.10 ± 2.39	Student’s t	-1.52	0.13
GC dose (mg/day)^1 ^M4	6.81 ± 1.88	7.39 ± 2.61	Student’s t	-0.97	0.33
GC dose (mg/day)^1 ^M6	5.81 ± 1.72	6.33 ± 2.38	Student’s t	-0.94	0.34
Δ GC dose (M0-M6)^1^	-2.55 ± 1.70	-2.77 ± 2.40	Student’s t	0.41	0.68

## Discussion

Our study demonstrated that a significant reduction in GC exposure could be achieved during the first six months following biologic therapy initiation in patients with RA, regardless of the treatment line. More than half of patients (31, 51.7%) achieved a GC dose of ≤5 mg/day, reflecting a substantial GC-sparing effect of biologic therapies in routine clinical practice. The mean GC dose decreased from 8.74 ± 1.93 mg/day at M0 to 6.07 ± 2.07 mg/day at M6. However, complete GC withdrawal remained uncommon, occurring in only one patient (1.7%). Baseline DAS28-CRP was significantly lower in patients who achieved a GC dose ≤5 mg/day at M6 than in those who remained on >5 mg/day (4.82 ± 0.77 vs. 5.36 ± 0.96, p = 0.02). The low rate of complete GC withdrawal observed in our study may be explained by the relatively short follow-up period, the long-standing nature of RA in our cohort, previous biologic exposure, and the high prevalence of comorbidities such as fibromyalgia. These findings highlight that although biologic therapies facilitate GC tapering, complete discontinuation remains challenging to achieve in a substantial proportion of patients. Nevertheless, the observed reduction in GC dose is clinically relevant, as it may reduce the risk of GC-related adverse effects and is consistent with current EULAR recommendations to minimize GC exposure whenever possible.

The challenges associated with GC withdrawal observed in our cohort have also been highlighted in the SEMIRA trial [11]. Although GC tapering was feasible in a substantial proportion of patients with well-controlled RA receiving biologic therapy, maintenance of low-dose prednisone (5 mg/day) was associated with better disease control and fewer disease flares compared with complete withdrawal. Overall, our findings are consistent with those of the SEMIRA trial, supporting the feasibility of GC dose reduction while highlighting the persistent challenges of achieving complete discontinuation despite effective biologic treatment.

Evidence from the international TOCERRA and PANABA collaborative studies further supports our observations. In these large international TOCERRA and PANABA collaborative cohorts, GC use decreased significantly following initiation of TNF inhibitors, tocilizumab, or abatacept. Nevertheless, the median time required to achieve complete GC withdrawal remained approximately two years or longer in most countries, emphasizing the persistent difficulties of complete GC withdrawal despite effective biologic therapy [13]. Similarly, complete withdrawal remained uncommon at M6 in our cohort despite a significant reduction in GC doses.

Additional evidence comes from the recent STAR trial, which compared two GC withdrawal strategies in patients with RA and low disease activity. Despite a structured tapering protocol and stable disease control, only 47% to 55% of patients achieved complete GC withdrawal after 12 months [14]. These results confirm that, even under optimized management conditions, successful GC withdrawal remains difficult to achieve.

Similar observations have been reported in real-world cohorts of patients receiving biologic therapy. In a study of patients with low disease activity treated with stable biologic or targeted synthetic DMARD therapy, 54% successfully achieved GC tapering. In contrast, complete prednisone discontinuation was observed in only 33% of cases. These findings further illustrate the gap between treatment recommendations and routine clinical practice and are consistent with the low rate of complete GC withdrawal observed in our study [15].

One possible explanation for the persistence of low-dose GC therapy in our cohort relates to the increased risk of flare associated with intensive tapering strategies. Adami et al. reported that tapering GCs to doses ≤2.5 mg/day or complete discontinuation was associated with a significantly increased risk of flare in patients receiving biologic therapy, whereas tapering to doses >2.5 mg/day was not associated with a significantly higher flare risk [16]. These findings suggest that maintaining low-dose GC therapy may contribute to disease stability in selected patients and may partly explain the limited rate of complete GC withdrawal observed despite significant improvement in disease activity.

The clinical relevance of the 5 mg/day prednisone threshold deserves particular attention. The GLORIA trial [12], conducted in elderly patients with RA, demonstrated that the addition of low-dose prednisolone (5 mg/day) resulted in improved disease control and reduced radiographic progression compared with placebo. However, these benefits were accompanied by an increased incidence of adverse events, particularly infections and other GC-related complications. These findings suggest that although a daily prednisone dose of 5 mg may contribute to maintaining disease control, it cannot be considered entirely risk-free. In our study, more than half of the patients achieved a GC dose ≤5 mg/day at M6, whereas complete GC withdrawal remained uncommon. Together, these findings highlight the clinical challenge of balancing optimal disease control with minimization of long-term GC exposure.

Our findings are further supported by the study of Spinelli et al. [17], which evaluated GC tapering and discontinuation in patients with RA treated with tofacitinib. The authors reported that a substantial proportion of patients were able to discontinue GCs as early as week 12 following treatment initiation, with approximately one-third achieving complete GC withdrawal during follow-up. These findings suggest that, despite the effectiveness of targeted therapies, complete GC withdrawal remains challenging in a considerable proportion of patients. This observation is consistent with our results, where a significant reduction in GC doses was achieved, whereas complete withdrawal was observed in only one patient.

Additional evidence supporting the GC-sparing effect of biologic therapies comes from the SPARE-1 study [18], which evaluated patients with RA treated with tocilizumab while receiving more than 5 mg/day of prednisone. After 12 months of treatment, 40% of patients achieved a GC dose below 5 mg/day without intensification of their disease-modifying therapy. However, complete GC withdrawal remained difficult to achieve, highlighting that dose reduction is often more feasible than full discontinuation. These findings further emphasize the persistent challenges associated with GC withdrawal despite effective control of disease activity with biologic therapies.

Strengths and limitations

To our knowledge, this is among the few longitudinal studies specifically evaluating the GC-sparing effect of biologic therapy in patients with RA in routine clinical practice in Morocco. The longitudinal design allowed for standardized data collection at predefined time points (M0, M4, and M6) and enabled assessment of GC dose evolution over time. Additionally, the inclusion of two biologic treatment groups (rituximab and anti-TNF agents) provided an opportunity to compare GC-sparing outcomes between different therapeutic classes. Furthermore, the concomitant assessment of disease activity (DAS28 and SDAI), inflammatory markers, pain intensity, and functional status strengthened the clinical relevance and interpretation of the observed results.

However, some limitations should be acknowledged. First, this was a single-center observational study with a relatively limited sample size, which may restrict the generalizability of the findings. Second, the follow-up period was limited to six months and therefore did not allow evaluation of the long-term sustainability of GC reduction or the maintenance of complete GC withdrawal. Finally, GC tapering strategies were not standardized. Additionally, no multivariable analysis was performed to identify independent predictors of successful GC tapering.

## Conclusions

Biologic therapies were associated with a significant GC-sparing effect in patients with RA. A progressive and significant reduction in GC doses was observed during the first six months of follow-up, with 31 patients (51.7%) achieving a daily GC dose ≤5 mg. Baseline DAS28-CRP was significantly lower in patients who achieved this GC dose threshold at M6.

Despite this favorable effect, complete GC withdrawal remained uncommon, occurring in only one patient (1.7%), highlighting the persistent challenges of corticosteroid discontinuation in patients with long-standing RA. This observation is consistent with previous studies showing that, although GC tapering is achievable in a substantial proportion of patients receiving biologic or targeted therapies, complete withdrawal often remains difficult to attain.

Overall, these findings suggest that reducing GC exposure to the lowest effective dose represents a realistic therapeutic goal for many patients with RA. Nevertheless, prospective multicenter studies involving larger patient populations and longer follow-up periods are needed to confirm these findings, identify predictors of successful GC withdrawal, and optimize tapering strategies while maintaining sustained disease control.
